# Atrial Fibrillation: Pathogenesis, Predisposing Factors, and Genetics

**DOI:** 10.3390/ijms23010006

**Published:** 2021-12-21

**Authors:** Marios Sagris, Emmanouil P. Vardas, Panagiotis Theofilis, Alexios S. Antonopoulos, Evangelos Oikonomou, Dimitris Tousoulis

**Affiliations:** 1 1st Cardiology Clinic, ‘Hippokration’ General Hospital, School of Medicine, National and Kapodistrian University of Athens, 11528 Athens, Greece; vardas.man@gmail.com (E.P.V.); panos.theofilis@hotmail.com (P.T.); alexios.antonopoulos@cardiov.ox.ac.uk (A.S.A.); boikono@gmail.com (E.O.); drtousoulis@hotmail.com (D.T.); 2 Department of Cardiology, General Hospital of Athens “G. Gennimatas”, 11527 Athens, Greece; 3 3rd Department of Cardiology, “Sotiria” Thoracic Diseases Hospital of Athens, University of Athens Medical School, 11527 Athens, Greece

**Keywords:** atrial fibrillation, pathogenesis, oxidative stress, predisposing factors, diets, Mediterranean diet, genetics

## Abstract

Atrial fibrillation (AF) is the most frequent arrhythmia managed in clinical practice, and it is linked to an increased risk of death, stroke, and peripheral embolism. The Global Burden of Disease shows that the estimated prevalence of AF is up to 33.5 million patients. So far, successful therapeutic techniques have been implemented, with a high health-care cost burden. As a result, identifying modifiable risk factors for AF and suitable preventive measures may play a significant role in enhancing community health and lowering health-care system expenditures. Several mechanisms, including electrical and structural remodeling of atrial tissue, have been proposed to contribute to the development of AF. This review article discusses the predisposing factors in AF including the different pathogenic mechanisms, sedentary lifestyle, and dietary habits, as well as the potential genetic burden.

## 1. Introduction

Over the past hundred years, atrial fibrillation (AF) is the arrhythmia that has been studied the most among all other heart rhythm disorders, leading to valuable conclusions [1]. The prevalence of AF ranges from 2% in the general population to 10–12% in those aged 80 and older [2]. It is the most common arrhythmia in humans, and incidence increases with advancing age [2]. According to the Global Burden of Disease, the estimated prevalence of AF is up to 33.5 million individuals, as it affects 2.5–3.5% of populations in several countries [3]. Atrial fibrosis has emerged as a significant pathophysiological component, with links to AF recurrences, resistance to medication, and complications [3]. Studies on the histological as well as electrophysiological aspects of the disease have led to its better understanding, improving the therapeutic possibilities and effectively, the quality of life of patients [1]. However, crucial questions regarding the formation and perpetuation of the disease remain unanswered. In this article, an update is presented on the emerging data connecting oxidative stress and inflammation to unfavorable atrial structural and electrical remodeling [4]. Moreover, it is epidemiologically proven that AF is correlated to several factors that either individually or in combination promote the initial development of the arrhythmia and the episodes that characterize the disease [5,6]. Undoubtedly, aging constitutes the primary factor responsible for the pathogenesis of the arrhythmia [5]. Additionally, arterial hypertension, obesity, diabetes mellitus and genetic factors have also been confirmed by the Framingham studies to be significant predisposing factors of the disease, while multiple dietary components seem to play a protective role reducing the occurrence of AF [7,8,9]. The present review summarizes the role of specific risk factors and pathophysiological mechanism in the development and perpetuation of the arrhythmia.

## 2. Fibrosis

Several mechanisms have been postulated to play a role in the development of AF, through both the electrical and structural remodeling of the atrial tissue. Among them, fibrosis has been studied thoroughly, confirming its significant role in this process.

Fibrosis refers to the increased deposition of extracellular matrix proteins in the myocardial interstitial tissue due to the excessive proliferation of fibroblasts in response to pathological conditions. Fibroblasts are responsible for the structural support and maintenance of the homogeneity of the cardiac tissue. During the fibrotic process, fibroblasts differentiate to myofibroblasts, cells that have been studied for their effect on reducing conduction velocity in the myocardium, promoting an arrythmogenic substrate [10].

Fibroses can been classified into two distinct types, reparative and interstitial fibrosis:

1. Reparative fibrosis refers to the replacement of necrotic myocardial cells by fibrotic tissue [9,11].
2. Interstitial fibrosis can be sub-classified into:
  (a) Reactive fibrosis, which indicates the deposition of extracellular matrix (ECM) in the interstitial and perivascular space without the replacement of the damaged cells [9,11];
  (b) Infiltrative interstitial fibrosis, which refers to the deposition of glycosphingolipids or insoluble proteins in the interstitial space, as seen in amyloidosis or Fabry disease respectively [12].

The two different types of fibrosis may coexist.

### 2.1. Cellular Mediators of Atrial Fibrosis

Several cellular subtypes have been investigated for their effect in the fibrotic process and the subsequent promotion of atrial fibrillation. Among them, fibroblasts have been established as the main cellular effectors of atrial fibrosis [13]. Fibroblasts are small, spindle-shaped cells of mesenchymal origin, accounting for 10–15% of all cardiac tissue cells. [14] They are metabolically active cells, regulating the synthesis and turnover of the ECM, thus preserving the architectural integrity of the cardiac tissue. Multiple communication pathways have been established between fibroblasts and cardiomyocytes, altering the latter’s electrophysiological properties. Under various pathological conditions and stress indicators, a phenotypic conversion of fibroblasts to alpha-smooth-muscle actin (αSMA) expressing myofibroblasts, takes place.

In detail, the activation and differentiation of local cardiac fibroblasts is dependent on multiple neurohumoral and mechanical profibrotic stress stimuli. Among the biochemical signals that have been identified to induce fibroblast differentiation, TGFβ has a prominent role in this process through both a canonical (SMAD-dependent) and non-canonical (SMAD-independent) pathway, which mediates the transcription of myofibroblast genes [15,16]. Additionally, angiotensin II (AngII) and endothelin 1 (ET-1), which bind to the G-protein-coupled receptors (GPCR) presented by cardiac fibroblasts, have been established as fibrotic mediators through the activation of a signaling cascade that promotes fibrotic gene transcription [17]. The activation and differentiation of fibroblasts is further enhanced when mechanical forces are applied that generate a more tensile and rigid matrix. The mechanisms that have been proposed to be responsible for the tension-based induction of myofibroblasts rely either on the activation of stretch-sensitive transient receptor potential (TRP) channels, which further activate factors such as TGFβ, or the force-mediated activation of p38 from the contractile signals of the cytoskeleton [18]. In conjunction with the aforementioned traditional fibroblast activation pathways, recent studies have brought to light significant mitochondrial, as well as cellular, metabolic components that promote the formation of myofibroblasts. Mitochondria act as key regulators in the fibroblast activation process by reducing their Ca^2+^ uptake in response to the profibrotic signals, a process that further enhances the cytosolic Ca^2+^ signaling pathway. Additionally, the profibrotic stressors induce the production of mitochondrial ROS, which activate factors such as p38 and ERK1/2, known for augmenting the transcription of fibrotic genes [19]. Lastly, various cellular metabolic functions have been highlighted over the past few years among the main drivers of myofibroblast formation. In particular, an increase in the rate of glutaminolysis in fibroblasts is considered crucial for their activation, while alterations in glycolysis with the subsequent increase in lactate production have been proposed as essential mechanisms for the promotion of the myofibroblast differentiation program [20]. Myofibroblast actions include recruiting inflammatory cells, promoting wound contraction, and secreting an excessive amount of ECM proteins such as collagen type I, III and IV; periostin; and fibronectin, leading to fibrosis [21,22].

In addition to fibroblasts, multiple inflammatory cells have been shown to be involved in the pro-fibrotic process. Studies have demonstrated the principal role that macrophages have in the regulation of fibrosis. Resident macrophages, originating from yolk sac-derived erythromyeloid progenitors (EMPs), populate the healthy myocardium, promoting its homeostasis. During the event of cardiac injury, multiple blood-borne monocytes infiltrate the myocardium and differentiate to macrophages [23]. Monocyte-derived macrophages express broad heterogeneity, enabling them to exert different functions, such as the production of multiple pro-fibrotic growth factors (IL-10, TGF-β, IGF-1, and PDGF), pro-inflammatory cytokines (IL-6, TNF-α, ROS), and proteases that contribute to matrix remodelling [23].

Likewise, following myocardial injury, T-cells populate the cardiac tissue in response to cytokine signalling. T-cells are then differentiated into either CD4+ (Th1, Th2) or CD8+ cytotoxic T cells, which exert distinct functions. In the immediate post-insult period, Th1 and CD8+ cells are the main residents of the myocardium [24]. These cells have been recognised for their anti-fibrotic functions, as they release mediators, such as IFN-γ and protein-10, which inhibit the action of the pro-fibrotic TGF-β. Additionally, INF-γ interferes with the activation of Th2 cells by impacting the production of IL4 and IL13 [24]. Progressing into the chronic injury period, Th2 cells overtake Th1 cells as the principal CD4+ cell phenotype in the myocardial tissue. In contrast to the latter, Th2 cells exhibit significant pro-fibrotic activity. This is performed mainly by secreting IL4 and IL13, molecules that stimulate collagen secretion either by enabling TFG-β or by recruiting monocytes in the lesion site [24].

**Figure 1:**
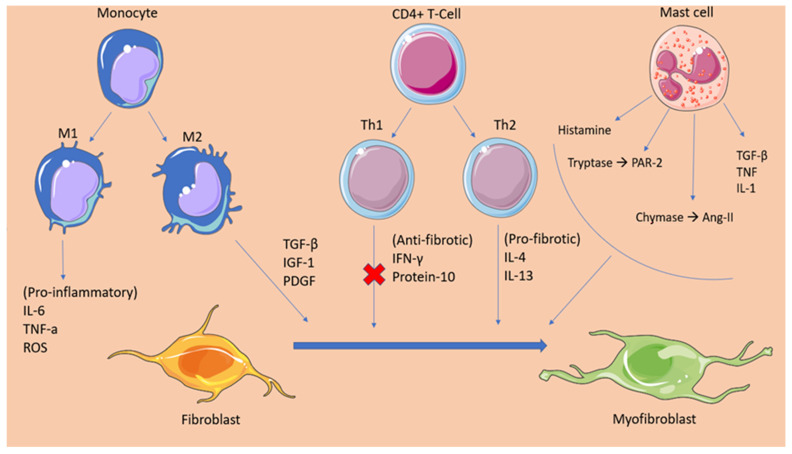
This graphical abstract summarizes the cellular mediators of atrial fibrosis. Following an insult, inflammatory mediators signal immune cells such as monocytes, CD4+ T-cells, and mast cells to infiltrate the atrial myocardium. These cells promote tissue fibrosis by secreting pro-fibrotic factors and regulatory molecules that enhance the activation and differentiation of fibroblasts to myofibroblasts. Additionally, the figure depicts the anti-fibrotic mediators that are secreted by Th1 cells in the early-insult stage and that are gradually overhauled by the products of pro-fibrotic Th2 cells. TGFβ, transforming growth factor beta; TNFα, tumor necrosis factor alpha; PDGF, platelet-derived growth factor; IL-1, interleukin 1; IL-4, interleukin 4; IL-6, interleukin 6; IL-10, interleukin 10; ROS, reactive oxygen species; IFNγ, interferon gamma; IGF-1, Insulin-like growth factor 1; Th1, t helper type 1; Th2, t helper type 2; PAR-2, protease activated receptor 2; Ang-II, angiotensin.

Another component of the innate immunity, mast cells, have established their role as modulators for cardiac fibrosis. Studies have demonstrated that, under conditions of cardiac ischemia and pressure overload, mast cells multiplicate and degranulate pre-formed inflammatory and fibrotic (e.g.,TGF-β1, TNF, IL-1) mediators. Mast cells present in the cardiac tissue represent the connective tissue phenotype and contain both chymase and tryptase. Shiota et al. conducted a study that identified a 5.2-fold increase in chymase activity in hamsters with chronic pressure-overloaded hearts [25]. Multiple studies have proven the pro-fibrotic effect of increased chymase activity in cardiac remodelling by promoting the formation of angiotensin-II [26,27,28]. The increased levels of tryptase in fibrotic hearts have been shown to mediate fibroblast proliferation and differentiation to myofibroblasts. The mechanism responsible has been attributed to the stimulation of protease activated receptor-2 (PAR-2) in fibroblasts and the subsequent phosphorylation of extracellular signal-regulated protein kinases 1 and 2 (ERK ½), which promotes the differentiation of fibroblasts to myofibroblasts [29]. Lastly, the role of histamine produced by mast cells has been thoroughly studied, establishing its significance in cardiac fibrosis. The detrimental role of histamine in cardiac fibrosis has been proven in an animal experiment, wherein a lack of histamine induced a response in H_2_-receptor-deficient mice and reduced myocardial apoptosis and fibrosis [30]. Nevertheless, multiple anti-inflammatory and anti-fibrotic mediators are also among the degranulation products of mast cells, raising controversy over the exact function of mast cells in the process of tissue remodelling [31] (Figure 1).

### 2.2. Fibrotic Mechanisms Inducing Atrial Fibrillation

Fibrosis has been established as a significant factor maintaining atrial fibrillation. There has been increased data associating the atrial remodelling induced by fibrosis with the promotion of AF. It has been proposed that the increased population of fibroblasts/myofibroblasts present in the fibrotic tissue and the increased deposition of ECM disrupt the myocardial bundles continuity, interfering with the gap-junction formation among cardiomyocytes. This event leads to conduction abnormalities, slowing conduction velocity and eventually forming unidirectional conduction blocks [32]. Moreover, as mentioned previously, myofibroblasts form communication channels with cardiomyocytes, altering their electrophysiological properties, giving rise to focal firing and re-entrant circuits.

Over the last 10 years, several clinical studies have been conducted to confirm the aforementioned mechanisms. Researchers from the Cardiovascular Research institute in Maastricht performed epicardial mapping in 24 patients with long-standing persistent AF, undergoing cardiac surgery, in an attempt to uncover the spatiotemporal characteristics of the fibrillatory process underlying the disease. The study confirmed the intra-atrial conduction disturbances with the presence of block lines running in parallel to the muscular bundles [33]. Additionally, a significant contribution in understanding the pathophysiology underlining the relationship between atrial fibrosis and arrhythmogenesis was made by Sebastien P.J Krul, et al., who studied the effect of interstitial fibrosis on conduction velocity [34]. Researchers obtained 35 atrial appendages during AF surgery and recorded the activation time as well as the longitudinal (CVl) and transverse (CVt) conduction velocity (CV). The results demonstrated that the thick interstitial fibrotic strands were directly associated with an increase in the longitudinal CV in contrary to the transverse CV, which was not affected [34]. However, a greater extent of transverse activation delay was observed because of the presence of activation block areas leading to a pattern of zig-zag conduction. This study points at the quality rather than the quantity of the fibrotic tissue as responsible for the formation of an arrhythmogenic substrate, with re-entry circuits enabling the perpetuation of atrial fibrillation [34]. To further verify the driver mechanisms of AF, Hansen et al. performed a simultaneous mapping of the sub-endocardial and sub-epicardial activation patterns, and then integrated these data to an MRI-produced atrial model, in an attempt to visualize the AF drivers. The researchers confirmed the presence of longitudinal conduction blocks in agreement with the epicardial mapping study and, in addition, proved that fibrosis due to cardiac diseases disrupts the myocardial architecture, promoting a structural substrate for re-entrant AF drivers [35].

## 3. Oxidative Stress

Over recent years, oxidative stress has been investigated as a potential essential mechanism in the development of AF. Reactive oxygen species (ROS) constitute the normal byproducts generated through the metabolism of oxygen. These molecules have been proven to have a multifaceted effect on the cells present in the heart tissue. Tahhan et al. recently revealed that the prevalence and incidence of AF were related to the redox potentials of glutathione (E_h_GSH) and cysteine, markers of oxidative stress. The study concluded that the prevalence of AF was 30% higher for each 10% increase in E_h_GSH, while the same alteration resulted in a 40% increase in the risk of incident AF [36]. The molecular processes underpinning atrial fibrillation development have been the subject of multiple clinical studies. Research evidence suggest that excessive ROS can directly affect ion channels and the propagation of action potential [37]. Hydrogen peroxide provokes trigger activity through the enhancement of late Na+ current, inducing early afterdepolarization (EAD) and delayed afterdepolarization (DAD). Moreover, ROS can induce a downregulation of the total Na+ current, an event that promotes the formation of reentry circuits. It is also worth mentioning that ROS can directly upregulate the L-type Ca^2+^ current and promote EADs by altering the intracellular calcium balance [37]. Recent experimental evidence suggests that the oxidation of ryanodine receptor 2 (RYR2) induces the intracellular release of Ca^2+^ from the sarcoplasmic reticulum, promoting the establishment of atrial fibrillation [38]. The generation of ROS in the myocardium has been attributed to many enzymatic sources. Among them, NADPH oxidase (NOX) has proven to have a critical role in the progress of AF. In studies performed in animal models, superoxide and H_2_0_2_ produced from activated NOX2 and NOX4 isoforms lead to myocyte apoptosis, fibrosis, and inflammation, which further promote atrial fibrillation perpetuation. One proposed mechanism through which ROS could exert their pro-arrhythmic function is by the oxidation of calmodulin-dependent protein kinase II (CaMKII) [39]. Oxidized CaMKII mediate the phosphorylation of the RYR2, leading to calcium overload and the formation of multiple wavelets triggering atrial fibrillation emergence [40]. In addition to the electrical remodeling stimulated by the mechanisms described, ROS have also been demonstrated to contribute to atria structural remodeling. Researchers from Slovakia showed that hydroxyl radicals can alter the myofibrillar protein structure and function, promoting myocardial injury and further contributing to the formation of a fertile substrate for the development of arrhythmias [5,41].

## 4. Inflammation

Inflammation has been linked to the onset and maintenance of atrial fibrillation, according to accumulating evidence. Inflammation contributes to the atrial remodelling involving both structural and electrophysiological alterations that form the basis for the disease. A large-scale prospective study involving 24,734 women participants investigated the association of inflammatory markers such as CRP, fibrinogen, and intercellular adhesion molecule 1 (sICAM-1) with the incidence of AF. The results suggested that inflammation is a strong indicator for the incidence of AF with the median plasma levels of the biomarkers being independently correlated with the development of the disease in patients [42]. That suggestion was further confirmed when scientists from Greece observed that the levels of high-sensitivity C-reactive protein (hs-CRP) are directly linked with the recurrence of AF after cardioversion and that the restoration of sinus rhythm (SR) resulted in a gradual decrease of hs-CRP [43], while Rotter et al. reported that CRP levels in individuals with AF declined following effective ablation [44,45]. Additionally, in a recent study, Yao C. et al. demonstrated that, in patients with atrial fibrillation, the activity of NLRP3 (NOD-, LRR-, and pyrin domain-containing protein 3) inflammasome in atrial cardiomyocytes was considerably enhanced. The upregulation of the NLRP3 inflammasome promotes the release of damage-associated molecular patterns (DAMPs), which lead to the activation of cardiac fibroblasts, cells that, as described earlier, are the main effectors of cardiac fibrosis [13].

Advances in the field of cardiology over the last years have led to the identification of many cellular and molecular mechanisms that suggest inflammation is responsible for the pathogenesis of AF. Under inflammatory stress, angiotensin II stimulates the production of proinflammatory cytokines (e.g., IL-6, IL-8, TNF-α) and the recruitment of immune cells. The role of AngII has also been established in the fibrosis and structural remodelling of the cardiac tissue through the activation of the MAPK-mediators of AngII/AT1R and the subsequent expression of the pro-fibrotic TGFβ1, which promotes fibroblast differentiation. Furthermore, increased pressure overload, as well as several gene polymorphisms in renin and angiotensin, mediate the formation of angiotensin II and the activation of angiotensin II receptors. Angiotensin II has been linked with the activation of NOX and the subsequent oxidation-related calcium-handling abnormalities, resulting in the electric remodelling of the atria. Additionally, NOX is a potent stimulator of the transcription factor nuclear factor-κB (NF-κB), which directly affects the sodium channel promoter regions, leading to a downregulation of the sodium channels and the promotion of AF mechanics [46,47]. The RAAS system mechanism lying behind AF development reflects the theory that atrial fibrillation begets atrial fibrillation. This notion can be justified by recent evidence suggesting that AngII not only causes inflammation but also that inflammation can promote AngII production through hs-CRP and TNF-a. These molecules, which are pronounced in inflammatory states, seem to have an upregulatory effect on the AT1R, further promoting this vicious cycle [48].

When associating inflammation with the occurrence of atrial fibrillation, it is important to mention the culprit of coronary artery disease in this phenomenon. Coronary heart disease has been associated with the development of atrial fibrillation through various mechanisms [49]. Among them, inflammation constitutes the most important determinant of atrial fibrillation presentation, second only to atrial infarction and the subsequent tissue fibrosis. Following the event of myocardial ischemia, local as well as systemic inflammation arises, which causes the release of various inflammatory factors such as IL-6 and CRP, which have been independently associated with the development of atrial fibrillation [50]. It has been proposed that IL-6 exerts its proarrhythmic effect by inducing atrial remodelling. Increased serum levels of IL-6 were associated by Psychari SN et al. with an increased left atrial size. The dilatation of the left atrium is believed to result from the stimulating effect of IL-6 on matrix-metalloproteinase-2 (MMP2), a protease that has been implicated in atrial remodeling [51]. Moreover, it has been demonstrated that inflammation induced by myocardial infarction can promote atrial remodeling through the activation of Toll-like receptors (TLR), factors of the innate immune system. Particularly, TLR 2 and TLR 4 mRNA expression is significantly enhanced in patients following MI, while elevated TLR-2 levels have been associated with increased left atrial size [52,53].

Of great importance when relating inflammation with AF, is the prothrombotic state present in the disease. A high CRP level has been related to the formation of thrombi in the left atrium [54]. Research has established the mechanisms of thrombogenesis in inflammation. During an inflammatory state, innate immune cells activation and the release of inflammatory ligands are upregulated. IL-2, IL-6, IL-8, TNF-a, and MCP-1 production is enhanced by the activated immune cells resulting in the synthesis of tissue factor (TF), von Willebrand factor (vWF), and P-selectin [55]. These molecules mediate platelet agglutination, as well as monocyte-endothelial cell attachment. This event combined with the endothelial damage induced in the atrium of a patient affected by AF severely increases the risk of thrombus formation [56,57,58] (Table 1).

## 5. Sedentary Lifestyle

Over the last decade, efforts have been made to prove the association between behavioral lifestyle and AF incidence [59,60]. Said, M. A et al. noted that a log-additive effect on the risk of developing cardiovascular diseases was present when the health habits and the individual genetic background were considered in a large population [60]. The American Heart Association (AHA) recently established the concept of the American Heart Association’s Life’s Simple 7 (LS7) metrics based on four healthy behavior metrics (non-smoking, normal weight, moderate physical activity, and a healthy diet) and three health factors (normal cholesterol, blood pressure, and fasting blood glucose [FBG]) [61,62]. Yang, Y. et al. and a MESA study showed that the subgroup with 3 to 7 ideal components from the optimal LS7 status had low risk of AF (57~59% reduced risk), while adherence to the optimal LS7 status reduced the risk even more [62,63]. As a result of extended, uninterrupted sitting, sedentary lifestyles cause negative alterations in blood insulin and glucose levels. Insulin resistance is linked to endothelial dysfunction due to a mismatch between the phosphatidylinositol 3-kinase (PI3K) and mitogen-activated protein kinase (MAPK) signaling pathways [64]. In an insulin-resistant condition, PI3K signaling is diminished, resulting in lower nitric oxide availability, but MAPK signaling is unchanged, resulting in increased endothlin-1 synthesis, endothelial cell death, and inflammation [64,65].

The way that weight reduction affects AF incidence and symptoms was analyzed in a randomized observational trial of 248 patients [66]. When compared to the control group, the intervention group lost significantly more weight (14.3 vs. 3.6 kg) and had significantly lower atrial fibrillation symptom burden scores, symptom severity scores, number of episodes (2.5 vs. no change), and cumulative duration (692-min decline and 419-min increase) [66]. As far as the benefits in cardiac remodeling are concerned, interventricular septal thickness (1.1 and 0.6 mm) and the left atrial area (3.5 and 1.9 cm) were reduced in the intervention and control groups, respectively. AF was associated with worse postoperative outcomes, in particularly in patients with carotid artery disease, revealing high stroke/death risk [66,67,68]. Previous research discovered that increased self-reported sitting is related with increased levels of adipokines, C-reactive protein and low-grade inflammation, a result that was independent of physical activity levels [64]. Increased reactive oxygen species (ROS) production inside the arterial wall may be responsible for vascular remodeling, promoting smooth muscle cell proliferation and generating endothelial dysfunction. The formation of ROS has been linked to sedentary lifestyle while being viewed as a significant component in the etiology of cardiovascular disease, notably due to the production of superoxide, which is related with endothelial function deficits and hypertension [69]. These findings suggest that targeting ideal cardiovascular health and weight reduction may limit the incidence and the severity of AF [66,68].

## 6. Dietary Habits

### 6.1. Alcohol-Resveratrol

Of interest is the effect of multiple dietary components in the pathogenesis or treatment of AF. High levels of alcohol intake were associated with increased occurrence of AF, while moderate consumption lead more males than females to AF [70]. More specifically, Larson et al. present the risk ratios among drinkers of <1 drink/week (12 g alcohol/drink), in a cohort study with 7245 AF cases. The results were the same regardless of the inclusion of binge drinkers: a hazard ratio of 1.01 for 1 to 6 drinks/week, 1.07 for 7 to 14 drinks/week, 1.14 for 15 to 21 drinks/week, and 1.39 for >21 drinks/week [70]. The findings note that even moderate alcohol consumption could potentially lead to AF. Low level of alcohol is still a debateable issue as a risk factor for AF in a large amount of studies. Ariansen I. et al. show that the consumption of up to ten alcoholic beverages per week appears to be harmless, while higher consumption constitutes a predisposing factor for AF [71,72].

Although reduced alcohol intake has to be a rational treatment target for patients with AF, resveratrol, a bioactive polyphenol, found in red wine, grapes, seeds, and peanuts has recently attracted scientific attention as a cardioprotective nutritional supplement due to its antioxidant and vascular effects [73,74]. In AF, resveratrol presents antiarrhythmic qualities as it potentially operates as an inhibitor of both intracellular calcium release and pathogenic signaling cascades, preventing calcium excess and maintaining cardiomyocyte contractile function. Attempts have been made to generate novel resveratrol derivatives for the treatment of arrhythmias [73,74].

### 6.2. Caffeine

Caffeine is a methylxanthine that has been considered a potential arrhythmiogenic substance. Caffeine is contained in coffee, tea, cola, and energy drinks and has neurohormonal and sympathetic nervous system effect. Previous studies note that moderate coffee consumption decreases the risk of heart failure, coronary heart disease, stroke, DM type 2, and all-cause mortality from cardiovascular disease, as compared to non-consumers [75,76]. As far as AF incidence is concerned, a dose-response is presented from 6 prospective cohort studies. Studies show a 11% reduction for low doses and 16% for high doses of caffeine consumption, while the AF incidence decreases by 6% for every 300 mg/d increment in habitual caffeine intake [77]. The risk of AF was greater in people who consumed fewer than two cups of coffee per day (12-oz cup of coffee ~140 mg of caffeine) compared to people with higher consumption. On the other hand, the likelihood of AF incidence declined when caffeine consumption exceeded 436 mg/day [77]. The Physicians Health Study highlights that men who reported drinking 1 to 3 cups of coffee every day have a decreased incidence of AF. Specifically, rare/never coffee consumption is associated with a hazard ratio for AF at around 1.0, ≤1 cup/week at 0.85, 2 to 4 cups/week at 1.07, 5 to 6 cups/week at 0.93, 1 cup/day at 0.85, 2 to 3 cups/day at 0.86 (0.76–0.97), and 4+ cups/day 0.96 [78]. An innovative study of Casiglia E. et al. observed 1475 unselected men and women and stratified them into three groups of caffeine intake, after genotyping for the −163C > A polymorphism of the CYP1A2 gene, regulating caffeine metabolism. With a larger caffeine intake, AF was considerably reduced in the third tertile (>320 mg/day) than in the first and the second, while no interaction was proven between slow caffeine metabolism and AF occurrence [79,80].

### 6.3. Mediterranean Diet

An increasing body of research suggests that the Mediterranean diet (Med-Diet) is useful in both the primary and secondary prevention of cardiovascular risk. This is achieved by the reduction of oxidation stress by the leading Med-Diet habits [81,82]. Patients with vascular events have lower glutathione peroxidase 3 (GPx3) levels compared to those without events; the Med-Diet favorably stimulates the antioxidant activity of GPx3 in AF, resulting in a reduced vascular event rate, while no differences regarding superoxide dismutase (SOD) activity have been found [83]. Pastori et al. showed significant reduction in AF’s vascular events in patients with adherence to the Med-Diet. More specifically, the group of patients with AF and higher adherence to Med-Diet had by far the fewest vascular events (5.3%) in comparison to the low-adherence group (23.4%) and the intermediate-adherence group (8.4%). These findings show that the downregulation of soluble NOX2-derived peptide (sNOX2-dp) and the decreased excretion of F2-isoprostanes (F2-IsoP) have a strong relationship with adherence to the Med-Diet and could lead to a reduction of cardiovascular events in AF patients, through an antioxidant effect [83]. Pignatelli P. and Pastori D. et al. present that platelet function in AF patients may be affected by increased adherence to the Med-Diet through the reduction of the urinary excretion of 11-dehydro-TxB2 or 11-dehydrothromboxane B2 produced from the breakdown of thromboxane A2- and the negative effect to gut-derived lipopolysaccharides (LPS), which may contribute to major adverse cardiovascular events [84,85].

### 6.4. Virgin Oil—Magnesium—Lean Fish

**Figure 2:**
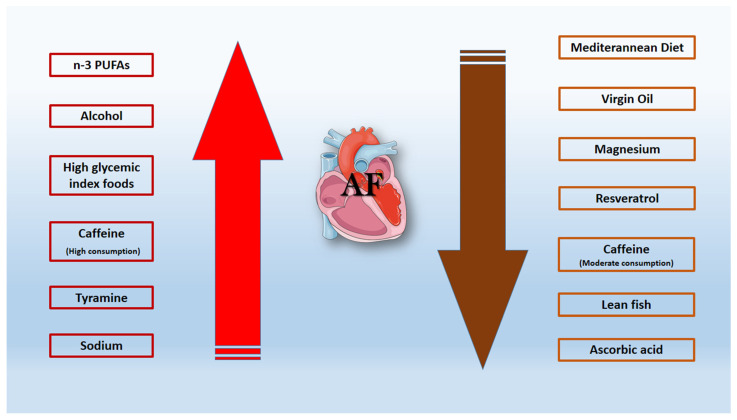
Graphical illustration of daily dietary habits that reduce (brown arrow) or increase (red arrow) the incidence of atrial fibrillation. n-3 PUFAs; polyunsaturated fatty acids.

A Mediterranean diet combined with extra virgin olive oil may lower the incidence of AF by the decrease of inflammatory markers—such as C-reactive protein or interleukin-6- and by its strong anti-oxidant effects. Magnesium has antiarrhythmic capabilities due to its tendency to modulate cardiac excitability by inhibiting calcium ion entrance into cells. According to a Mendelian randomization research, genetically greater blood magnesium levels may be related with a lower incidence of AF. This observation may have therapeutic implications because blood magnesium levels can be increased by supplementation and dietary recommendations,—boosting intake of green leafy vegetables—and intravenous administration [86,87]. As far as the effect of dietary intake of saturated fatty acids on the development of AF is concerned, when total n-3 polyunsaturated fatty acids replaced dietary saturated fatty acids, there was a slight rise in AF occurrences in males but not in women. Replacing saturated fatty acids with monounsaturated or long chain polyunsaturated -n-6 polyunsaturated- fatty acids was not associated with the risk of AF [88,89]. In the same pattern, the intake of total fish, fatty fish (herring/mackerel and salmon/whitefish/char), and long-chain omega-3 polyunsaturated fatty acids has no contribution in the occurrence of AF. In contrast, the group of patients who consume lean fish (cod/saithe/fish fingers) in a frequency of ≥3 servings/week presents a lower risk of AF than the group of never consumers [90,91] (Figure 2).

## 7. Genetic Factors

Over the last decade, the identification of genes related to AF is a domain that has garnered much media and scientific attention. Ion-channel mutations provide important information on the processes driving AF; therefore, many methodologies and classic Mendelian genetics have been utilized to determine the potential family foundation. As far as the K^+^ channel genes are concerned, the genes whose mutations increase the risk of AF occurrence are ABCC9 (I KATP), HCN4 (I f), KCNA5 (I Kur), KCND3 (I Ks), KCNE1 (IKs), KCNE2 (IKs), KCNE3 (IKs), KCNE4 (IKs), KCNE5 (IKs), KCNH2 (IKr), KCNJ2 (I K1), KCNJ5 (I KAch), KCNJ8 (I KATP), KCNN3 (IAHP), and KCNQ1 (IKs) [92]. The underlying mechanism is the higher K^+^ current reducing refractoriness and encouraging re-entry while decreasing automaticity [2]. It has been found that rare mutations in the gap junctional protein-coding gene GJA5 and in the nuclear pore complex (nucleoporin) Nup155 could cause AF and sudden death even at a young age. These cases of AF are likely to be caused by the re-entry mechanism. Equally important is the suggestion that loss-of-function mutations delay repolarization and promote Ca^2+^ mediated after depolarization triggers AF. The above-mentioned phenomenon could be caused by: (a) the variants SCN1B, SCN2B, SCN3B, SCN4B, SCN5A, and SCN10A of Na+ channel genes; (b) junctophilin mutation (E169K), which was found to enhance the RyR2 Ca^2+^ leak, leading to the juvenile onset of AF; (c) a single nucleotide polymorphism (SNP) in CASR, which encodes a Ca^+2^-sensing receptor that detects extracellular calcium ion levels and regulates calcium homeostasis [92]. The AFGen research uncovered 17 distinct susceptibility signals for AF at 14 different genetic locations; these include KCNN3, PRRX1, CAV1, SYNE2, C9orf3, HCN4, and MYOZ1 [2,92,93].

Various studies attempt to analyze the frequencies of single-nucleotide polymorphisms (SNPs) in genes whose protein products are involved in the pathogenesis of AF. Genome-wide association (GWAS) in the Japanese population identified that rs2200733, rs10033464 (located in the PITX2), and rs6584555 (located in the NURL1) were associated with AF [94]. In previous studies in Japan, six more loci were associated with AF: at 1q24 in PRRX1 (rs593479), 4q25 near PITX2 (rs2634073), 7q31 in CAV1 (rs1177384), 10q25 in NURL1 (rs6584555), 12q24 in CUX2 (rs649002), and 16q22 in ZFHX3 (rs12932445) [95]. The most significant finding was revealed in the study of Low S.K et al., in which different genetic factors lead to AF between Japanese and European population. Variants of KCND3, PPFIA4, SLC1A4-CEP68, HAND2, NEBL, and SH3PXD2A genes detected with five to six new loci differing between the two populations [96]. Korea Genome Epidemiology Study found two novel genetic loci on chromosomes 1q32.1/PPFIA4 (rs11579055) and 4q34.1/HAND2 (rs8180252), which were associated with the early-onset of AF. The loci on chromosome 4 has association with a previously proven gene in a European population. The found loci encode proteins involved in cell-to-cell communication, hypoxia, or long non-coding RNA [97].

Of scientific interest are the results of the GWAS on the variants of the transcription factor PITX2 [98,99]. The secretive protein is expressed in the adult left atrium, and, in early life, it is responsible for the regulation of the right–left differentiation of the embryonic heart, thorax, and aorta. The p.Met207Val variant produces a 3.1-fold increase in PITX2c transactivation activity in HeLa cells when compared to the wild-type equivalent. When the variant was expressed in contribution with wild-type PITX2c, an increase of arrhythmogenic mRNA levels of KCNH2 (2.6-fold), SCN1B (1.9-fold), GJA5 (3.1-fold), GJA1 (2.1-fold), and KCNQ1 in the homozygous form (1.8-fold) was revealed [98,100]. These genes encode for the IKr channel α subunit, the β-1 Na+ channel subunit, connexin 40, connexin 43, and the IKs channel α subunit, respectively [98,99,100]. Recent studies reveal that miRNAs can influence gene expression in hypertrophy and arrhythmia, as well as the numerous genes implicated in AF, making them viable molecular targets that may give greater clinical assistance. miR-21 and miR-133 seem to be involved in the structural remodeling of the atrium via enhanced fibrosis [101]. Differences in concentrations of miRNAs such as miR-21 between serum plasma and atrial tissue have been observed [101]. Studies have shown that miR-133b, miR-328, and miR-499 functionally control the ion regulating the activity of Ca^2+^ and K^+^ channels [102]. Their concentrations were higher in the bloodstream of patients with acute new-onset AF and chronic AF rather than those without or with well-controlled AF [103,104]. Consequently, it is necessary to perform further studies on the strong genetic background of AF and the early detection of miRNA polymorphisms. SNPs can also improve the diagnosis and management of the AF patients as potential biomarkers (Table 2).

## 8. Prevention-Conclusions

Physicians might always estimate the AF risk for patients with burdened health profile (age, hypertension, diabetes, obesity etc.) via the multiple scales that have been introduced. Obesity, excessive alcohol use, and obstructive sleep apnoea are all known to contribute to unfavourable LA remodelling and AF risk [105,106]. As such, lifestyle and dietary modifications including weight loss, alcohol reduction, and cardiometabolic risk factor management would be a cornerstone for AF prevention [105,107]. The medical prescription of medications other than anti-inflammatory agents, such as angiotensin-converting enzyme inhibitors, angiotensin receptor blockers, and aldosterone antagonists, can all help to reduce LA enlargement, atrial fibrosis, and TGF-β indicators, as well as atrial dysfunction. These are the most widely used drugs for AF and have to be considered for patients with a history of heart failure [108,109]. The novel SGLT-2 inhibitors reveal beneficial effects in systolic heart failure included improved cardiac energy metabolism, the prevention of inflammation, oxidative stress, adverse cardiac remodelling, less LA enlargement, fibrosis, atrial mitochondrial dysfunction, inflammation, and AF inducibility [110].

As far as the recognition of potential paroxysmal AF is considered, new strategies with smart watches and other devices can detect events better than a traditional 24-h ambulatory ECG recording [111,112]. Rapid progress has been made in identifying the genetic basis for this common condition. For individuals with a remarkable family history of AF or cardiomyopathy, DNA sequencing for potential genetic loci that are associated with AF would be beneficial [113]. The correction of unfavourable stressors can result in decreased atrial size and a reduction of electrophysiological anomalies in a scenario of established pathological atrial enlargement, owing to continuous increases in volume or pressure load. As such, clinicians’ high awareness of the field is the key point for the early identification of AF events, while the development of early prevention strategies and screening programs can be organized for patients with poor medical status [111].

Based on the above facts, it has become clear that fibrosis, inflammation, and oxidative stress, as well as behavioural and genetic factors, contribute decisively to the development of atrial fibrillation. Undoubtedly, the aforementioned risk factors, primarily the evolving atrial myopathy, form a fertile substrate for the establishment of anisotropic conduction properties in the atrial myocardium, the fragmentation of the electrical activity, and eventually, the development of atrial fibrillation.

## Figures and Tables

**Table 1:** Differences in concentrations of inflammatory proteins in patients with and without atrial fibrillation.

Protein	Protein Serum Levels Difference	Atrial Tissue Levels Difference	Predictor for AF
**CRP**	NA	NA	Yes
**MCP-1**	+	+	No
**MPO**	NA	+	No
**TGF-β**	NA	+	No
**TNF**	NA	+	No
**HSP-27**	+	+	NA
**HSP-70**	-	-	NA
**IL-1**	NA	NA	NA
**IL-6**	NA	+	No
**IL-8**	+	+	NA
**IL-10**	NA	+	NA

Abbreviations: AF = atrial Fibrillation, IL = interleukin, CRP = C-reactive protein, TNF = tumor necrosis factor, HSP = heat shock protein, TGF = transforming growth factor, MPO = myeloperoxidase, MCP-1 = monocyte chemoattractant protein, NA = not applicable, (+) = There is a difference in concentration; (-) = There is no difference in concentration.

**Table 2:** Genetic mutations that are implicated in atrial fibrillation.

Gene of	Polymorphism-Mutation	Action
**ABCC9 (I KATP)** **KCNA5 (I Kur)** **HCN4 (I f)** **KCND3 (I Ks)** **KCNE1 (IKs)** **KCNE2 (IKs)** **KCNE3 (IKs)** **KCNE4 (IKs)** **KCNE5 (IKs)** **KCNH2 (IKr)** **KCNJ2 (I K1)** **KCNJ5 (I KAch)** **KCNJ8 (I KATP)** **KCNN3 (IAHP)** **KCNQ1 (IKs)**	Potassium (K^+^) channel genes	The increased K^+^ current abbreviates refractoriness and promotes re-entry, while tending to reduce automaticity
**SCN1B** **SCN2B** **SCN3B** **SCN4B** **SCN5A** **SCN10A**	Sodium (Na^+^) channel genes	Delay repolarization and promote Ca^+2^ mediated after depolarization
**GJA5**	Mutations in the gap junctional protein	Re-entry mechanism
**NUP155**	Nuclear pore complex (nucleoporin) Nup155	Re-entry mechanism
**E169K**	Junctophilin mutation	Delay repolarization and promote Ca^+2^ mediated after depolarization enhancing RyR2 Ca^+2^ leak
**CASR**	rs1801725	Delay repolarization and promote Ca^+2^ mediated after depolarization
**PITX2**	rs2200733 rs10033464 rs2634073	PITX2 deficiency results in electrical and structural remodelling
**NURL1**	rs6584555 rs6584555	Undefined
**PRRX1**	rs593479	Undefined
**CAV1**	rs1177384	Undefined
**CUX2**	rs649002	Undefined
**ZFHX3**	rs12932445	Undefined

## Data Availability

Not applicable.
